# Metformin and glucose starvation decrease the migratory ability of hepatocellular carcinoma cells: targeting AMPK activation to control migration

**DOI:** 10.1038/s41598-019-39556-w

**Published:** 2019-02-26

**Authors:** Anabela C. Ferretti, Florencia Hidalgo, Facundo M. Tonucci, Evangelina Almada, Alejandro Pariani, María C. Larocca, Cristián Favre

**Affiliations:** Institute of Experimental Physiology, CONICET, School of Biochemical Sciences, University of Rosario, Rosario, Argentina

## Abstract

Hepatocellular carcinoma (HCC) is a highly metastatic cancer with very poor prognosis. AMP activated kinase (AMPK) constitutes a candidate to inhibit HCC progression. First, AMPK is downregulated in HCC. Second, glucose starvation induces apoptosis in HCC cells via AMPK. Correspondingly, metformin activates AMPK and inhibits HCC cell proliferation. Nevertheless, the effect of AMPK activation on HCC cell invasiveness remains elusive. Here, migration/invasion was studied in HCC cells exposed to metformin and glucose starvation. Cell viability, proliferation and differentiation, as well as AMPK and PKA activation were analyzed. In addition, invasiveness in mutants of the AMPKα activation loop was assessed. Metformin decreased cell migration, invasion and epithelial-mesenchymal transition, and interference with AMPKα expression avoided metformin actions. Those antitumor effects were potentiated by glucose deprivation. Metformin activated AMPK at the same time that inhibited PKA, and both effects were enhanced by glucose starvation. Given that AMPKα(S173) phosphorylation by PKA decreases AMPK activation, we hypothesized that the reduction of PKA inhibitory effect by metformin could explain the increased antitumor effects observed. Supporting this, in AMPK activating conditions, cell migration/invasion was further impaired in AMPKα(S173C) mutant cells. Metformin emerges as a strong inhibitor of migration/invasion in HCC cells, and glucose restriction potentiates this effect.

## Introduction

Hepatocellular carcinoma (HCC) is a rather frequent and much more aggressive cancer, mainly due to its feature of developing intra and extrahepatic metastasis at an extremely rapid rate^1^. Accumulation of genetic and microenviromental changes take place in hepatocytes during chronic inflammation associated to a basal liver disease in 90% of HCC patients, and this scenario promotes malignant transformation from early dysplastic to multiple and genetically-heterogeneous nodules^2^. Despite administration of surgical or current pharmacological treatment, most people diagnosed with HCC die within two years of being diagnosed, and this statistics positions HCC as the second cause of cancer death worldwide^2,3^. Elucidation of the mechanisms controlling cell proliferation and, specially, migration constitutes a main issue for understanding the bases of the disease and hence for foreseeing therapeutic strategies to limit its development.

In the last years, AMP activated kinase (AMPK) signaling was demonstrated to be involved in HCC etiology and has become a promising therapeutic target^4–7^. In fact, AMPK activity is significantly decreased in tumor compared with non-tumor region, and this downregulation is associated with worst HCC prognoses^4,6^. AMPK consists of a heterotrimer of catalytic (α), regulatory (β), and activation (γ) subunits, which response to energy stress in most tissues and cell types. Upon activation, AMPK enhances fatty acids and glucose oxidation and inhibits protein biogenesis thus leading to the restitution of ATP levels^8^. In addition, AMPK signals cell cycle arrest and survival regulation in tumor cells^9–11^. Moreover, even when it has not been as well characterized, AMPK activation can also affect cell motility and hence it can decrease the metastatic capacity of cancer cells^12–14^. We have recently demonstrated that AMPK is the key kinase pathway that controls cell death in HCC cells undergoing glucose restriction: AMPKα silencing in HCC cells prevents both cell cycle arrest and apoptosis induced by glucose starvation^15^. However, scanty information exists regarding the involvement of AMPK signaling in HCC cell migration. Besides the allosteric effect of AMP, activation of AMPK during nutritional stress requires phosphorylation of Thr172 residue of AMPKα by LKB1^8^. AMPK activation can be negatively regulated by phosphorylation of different regulatory residues by PKA and/or AKT^16–18^. Our previous findings indicated that Ser173 phosphorylation by PKA reduces phospho-AMPKα(T172) levels and prevents apoptotic activation in HCC cells subjected to nutritional stress^15^.

Metformin, an antidiabetogenic drug which in recent years has entered into the limelight of promising anticancer drugs^19^, is a bonafide AMPK activator. Metformin activates AMPK via affecting mitochondrial respiration complex I and AMP/ATP ratio^20^, as well as by favoring LKB1 activation^21,22^. Furthermore, it has been shown that metformin can also indirectly activate AMPK by inhibiting PKA and therefore decreasing AMPKα(S173) phosphorylation^23^. Recent studies showed that metformin diminishes proliferation in HCC cells *in vitro* or in xenotransplanted nude mice^4^. Because of its antiproliferative effects, metformin is nowadays being studied for cancer therapy in diverse clinical trials. Nevertheless, AMPK participation in the regulation of HCC cell migration and metformin putative actions on this pathway remain elusive.

We hypothesize that AMPK signaling can inhibit HCC cell migration and that the extent of this effect depends on AMPK activation effectiveness in each cellular context. In this study, we aimed to analyze migratory capacity in HCC derived cells treated with metformin and combined with glucose starvation condition. We presented strong evidence supporting that metformin exerted a considerable antimigratory effect in HCC cells which was potentiated by glucose restriction. Results on the migratory response of HCC cells with non phosphorylatable mutation of S173 residue of AMPKα were also analyzed.

## Results

### Metformin decreases migration in two HCC cell lines with different migratory behavior

**Figure 1:**
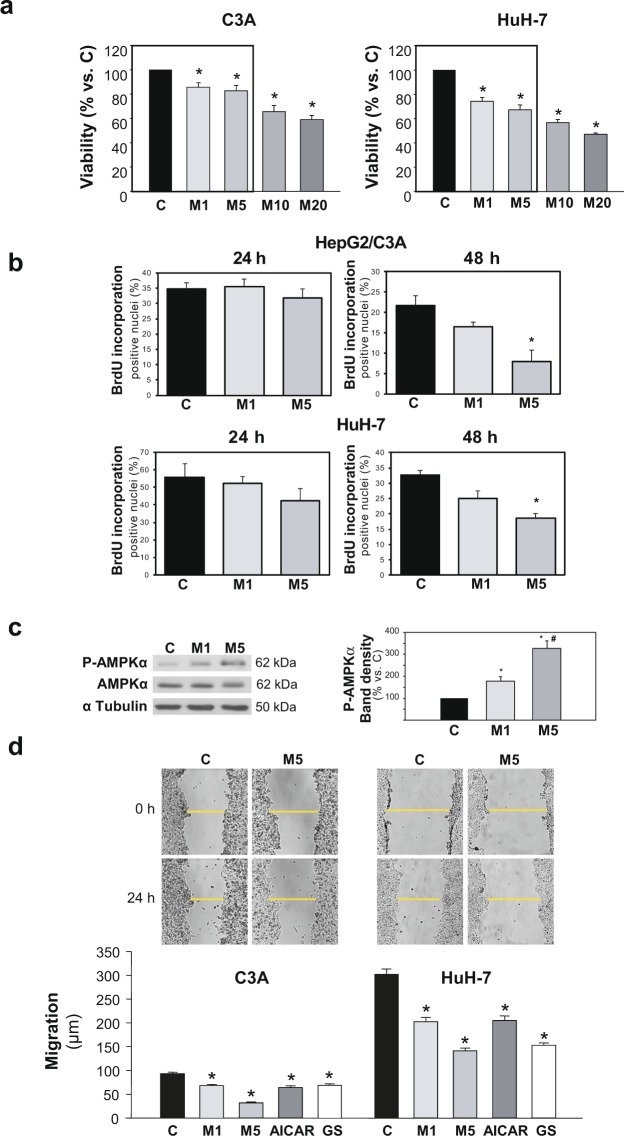
Metformin, from low concentrations, inhibits migration in HCC cells. (**a**) HCC cells were incubated for 24 h in complete DMEM alone (C), or in the presence of 1–20 mM metformin (M), and cell number was evaluated by MTT assay. Bars represent the mean cell viability expressed as percentage of C ± SEM of 4 independent experiments. (**b**) HCC cells were incubated for 24 or 48 h in complete DMEM alone (C), or in the presence of 1 (M1) or 5 mM (M5) metformin and cell proliferation was evaluated by BrdU incorporation assay. Bar represents the mean percentage of positive nuclei counted ± SEM. The assay was evaluated for 3 independent experiments (**c**) HepG2/C3A cells were incubated with complete DMEM (C), or in the presence of 1 (M1) or 5 mM (M5) metformin. P-AMPKα(T172) and AMPKα protein levels were detected in cell lysates after 24 h. α Tubulin was used as loading control. Selected lanes for each detection are in their original order and correspond to the same gel, and they are shown after cropping, aligning and separating them by white space. Full-length blots are available in Supplementary Dataset. Immunoblots show an experiment representative of 3 independent experiments. Bars represent P-AMPKα band densities relative to the corresponding α Tubulin band densities and are expressed as % of the control. *P < 0.05 *vs*. C, #*P* < 0.05 *vs*. M1. (**d**) Confluent HCC cells were subjected to scratch wounding (0 h) and incubated in complete DMEM alone (C), or in the presence of 1 (M1) or 5 mM (M5) metformin, or 1 mM AICAR (AICAR), or glucose starved by incubation in no-glucose DMEM (GS). Images illustrate wound gaps at 0 h and 24 h. Bars represent the mean distance migrated by the “wound front”. Values represent the mean ± SEM from 18 fields for an experiment representative of 3 independent experiments. *P < 0.05 *vs*. C.

HepG2/C3A and HuH-7 cell number after 24 h metformin incubation at a rank of concentrations most frequently used for cancer cell treatment was screened by MTT assay (Fig. 1a). Remarkably, 1 and 5 mM metformin led to mild (15–30%) decreases in total viable cells. The higher concentrations of 10 and 20 mM led to decreases in cell number which surpassed 50%. Proliferation rates in both cell lines subjected to metformin treatment at the lower concentrations were evaluated by BrdU incorporation assay: After 24 h treatment neither 1 mM nor 5 mM metformin induced any significant change in BrdU incorporation. On the other hand, after 48 h treatment, 1 mM metformin showed only a tendency to elicit an antiproliferative effect, although 5 mM metformin significantly decreased the number of positive nuclei (Fig. 1b).

In turn, the effect of metformin at these lower concentrations on 2D cell migration was evaluated by wound healing assays. The effects of two different AMPK activating conditions, AICAR and glucose deprivation, were also analyzed and compared using the same assay (Fig. 1d). Metformin significantly inhibited cell migration in a dose dependent manner in HepG2/C3A and HuH-7 cells (Fig. 1d). This effect was similar in both cell lines, regardless of their different migratory efficiencies, and it was in accordance with the extent in AMPK activation, which was mild with 1 mM metformin (+77%) and greater with 5 mM metformin (+228%) (Fig. 1c). In fact, the distance advanced by the cells 24 h after wounding was reduced by around 30% or 60% of the control group when cells were treated with metformin at 1 or 5 mM, respectively (Fig. 1d). The velocity of migration of individual cells in the wound front was also estimated by time-lapse studies. These results see Supplementary Fig. 1 corroborated that the effect of metformin on the migratory function detected by wound healing assay as a global measure of collective migration was similar to that observed by single-cell tracking.

Taking together, these results suggested that AMPK activation entailed antimigratory effects in HCC cells, and that 1 mM metformin was already effective in inhibiting the cell migratory ability, whilst only subtly induced citotoxicity.

### Metformin effects on transwell migration/invasion assay in HCC cells

**Figure 2:**
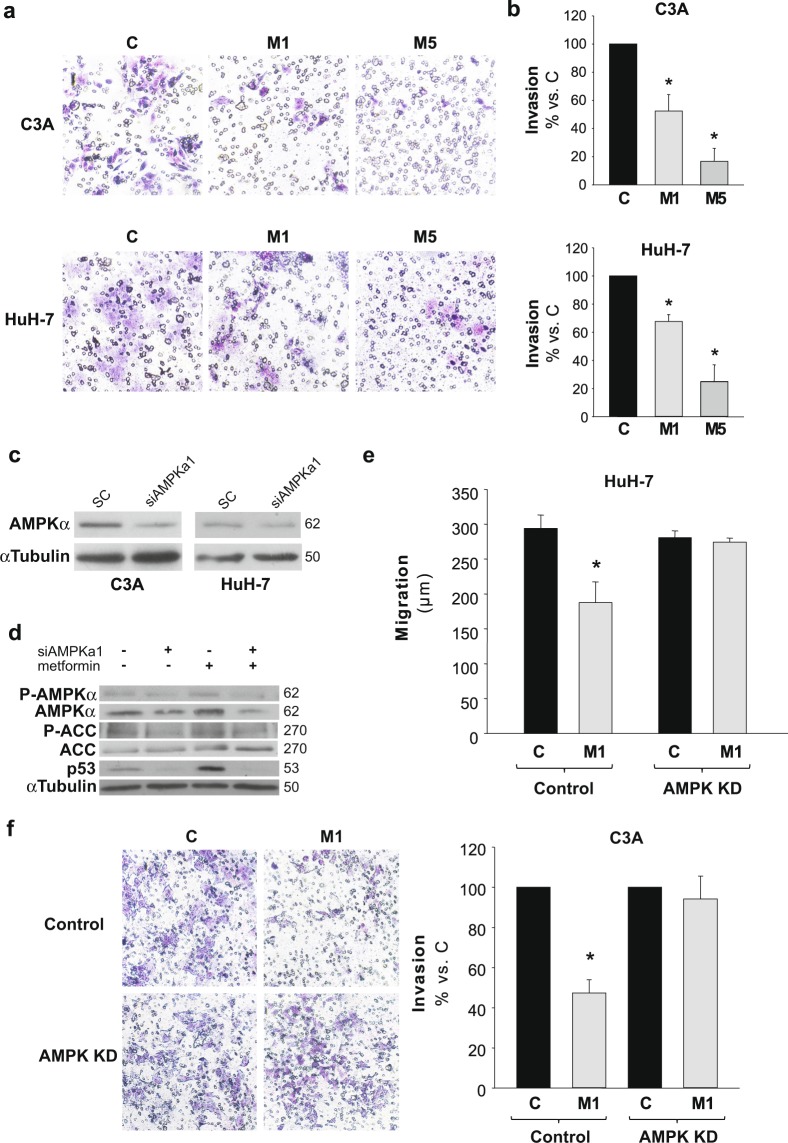
Metformin decreases invasiveness in HCC cells and AMPK knock down blocks metformin inhibition of cell migration/invasion. HCC cells were plated onto the upper compartment of Matrigel-coated filters of transwell chambers and incubated with complete DMEM alone (C) or in the presence of 1 (M1) or 5 mM (M5) metformin. After 48 h, cells that had migrated to the bottom of the filter were stained using toluidine blue, photographed and quantified. (**a**) Representatives images of cells that invaded the lower chamber are shown. (**b**) Quantitative data are shown as percentages of C. Bars indicate the mean ± SEM of 3 independent experiments. (**c**) HCC cells were transfected with AMPKα1 targeted (AMPK KD), or scrambled (Control) siRNAs. AMPKα expression was analyzed 72 h post-transfection by immunoblotting in Control and AMPK KD C3A and HuH-7 cells. α Tubulin was used as loading control. Selected lanes for each detection are in their original order and correspond to the same gel, and they are shown after cropping, aligning and separating them by white space. Full-length blots are presented in Supplementary Dataset. Immunoblots show an experiment representative of 3 independent experiments. (**d**) HepG2/C3A cells were transfected with AMPKα1 targeted or scrambled siRNAs and cultured for 48 h, after then additional 24 h treatment with or without 1 mM metformin was performed. P-AMPKα(T172), AMPKα, P-ACC, ACC and p53 were detected by immunoblotting. α Tubulin was used as loading control. Selected lanes for each detection are in their original order and correspond to the same gel, and they are shown after cropping, aligning and separating them by white space. Full-length blots are presented in Supplementary Dataset. Immunoblots show an experiment representative of 3 independent experiments. (**e**) 48 h post-transfection AMPK KD and Control HuH-7 cells were subjected to scratch wounding (0 h) and incubated with complete DMEM alone (C), or in the presence of 1 mM metformin (M1) for 24 h. Data were obtained as indicated in Fig. 1b. (**f**) 24 h post-transfection AMPK KD and Control C3A cells were plated in transwell chambers as detailed in **a** and incubated with complete DMEM alone (C), or in the presence of 1 mM metformin (M1) for 48 h, and processed as indicated in **a**. *P < 0.05 *vs*. C.

To further characterize the effects on cell migration/invasion, we performed a 3D cell migration/invasion assay by analyzing the ability of cells to migrate through a matrigel coated membrane. Figure 2a depicts typical captures of matrigel coated transwells after the different treatments. Cell invasiveness decreased, approximately, to 40 and 60% of control levels in C3A and HuH-7 cells, respectively, when treated with 1 mM metformin, and to 20% in the presence of 5 mM metformin (Fig. 2b). Collectively, these results showed that metformin was a potent inhibitor of migration/invasion in HCC derived cells.

### Silencing of AMPK suppresses metformin antimigratory and anti-invasive effects

The expression of AMPKα was diminished with specific siRNAs (AMPK KD) in HepG2/C3A and HuH-7 cells (Fig. 2c). Phosphorylation of AMPKα (Thr172) and ACC(Ser78/80) was detected in AMPKα silenced cells with or without 1 mM metformin in order to corroborate the involvement of AMPK signaling. We found that metformin-induced phosphorylations of AMPKα and ACC were abrogated in AMPK KD cells (Fig. 2d).

In addition, given that during energetic stress p53 status in hepatic cells seems to be tightly regulated by AMPK activity^24^, together with the fact that we previously observed increases of the p53 transactivated proteins p21 and Puma after AMPK activation by glucose starvation in HCC cells^15^, we decided to evaluate p53 levels in control and AMPK silenced HCC cells treated with metformin. Metformin entailed an augment of total p53 and this putative stabilization was dependent on AMPK. In fact, negligible amounts of p53 were observed in AMPK KD cells (Fig. 2d).

Migration and invasion assays were performed in control and AMPK KD cells in the presence of metformin. The interference of AMPKα expression abrogated the inhibition of migration (Fig. 2e) and invasion (Fig. 2f) elicited by 1 mM metformin in HCC cells. Besides, migration studies carried out in the p53-null cell line Hep3B demonstrated no inhibitory effects of metformin See Supplementary Fig. 2. These results suggested that an intact AMPK-p53 axis was necessary to observe the antimigratory effects of metformin in HCC cells.

### Metformin promotes acquisition of differentiated epithelial phenotype

**Figure 3:**
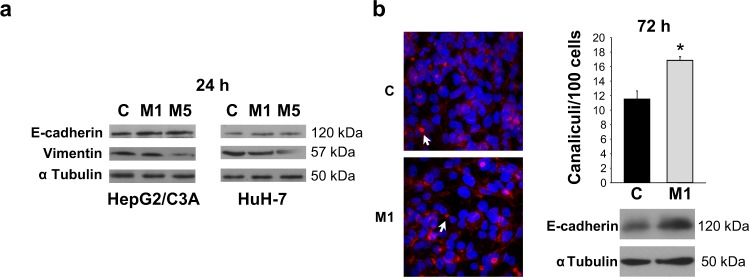
Metformin favors the acquisition of a differentiated epithelial phenotype in association with increased E-cadherin and reduced vimentin levels. (**a**) C3A and HuH-7 cells were incubated in complete DMEM alone (C) or in the presence of 1 mM (M1) or 5 mM (M5) metformin for 24 h and E-cadherin and vimentin were detected in cell lysates. α Tubulin was used as loading control. Selected lanes for each detection are in their original order and correspond to the same gel, and they are shown after cropping, aligning and separating them by white space. Full-length blots are available in Supplementary Dataset. Immunoblots show an experiment representative of 3 independent experiments. (**b**) C3A cells were incubated for 72 h in complete DMEM alone (C) or in the presence of 1 mM metformin (M1). Afterwards, cells were fixed and stained to visualize actin cytoskeleton and nuclei, and canalicular structures were quantified. Arrows indicate representative canaliculi. Bars represent the mean number of canaliculi per 100 cells. Values represent the mean ± SEM from 10 fields for an experiment representative of 3 independent experiments. Below, E-cadherin was detected in the corresponding cell lysates from each experimental group. α Tubulin was used as loading control. Immunoblots show an experiment representative of 3 independent experiments. *P < 0.05 *vs*. C.

Expression of epithelial and mesenchymal markers was evaluated. E-cadherin levels were significantly increased whilst vimentin levels were significantly decreased after being treated with metformin, at 1 or 5 mM, for 24 h (Fig. 3a). In spite of their tumor origin, HepG2/C3A cells can develop pseudocanaliculi, which resemble the epithelial structures typical of differentiated hepatocytes^25^. Considering that cell migration depends on changes in polarity associated to loss of epithelial features, cell differentiation was evaluated as the extent of formation of pseudocanaliculi in HepG2/C3A cells. In accordance with its antimigratory effect, 1 mM metformin increased the number of these canalicular structures in HCC cells (Fig. 3b). In association with this increase in differentiation, E-cadherin protein levels in these cells kept significantly augmented after 72 h treatment with 1 mM metformin (Fig. 3b). Altogether, these results indicated that metformin inhibited epithelial-mesenchymal transition (EMT) in HCC cells.

### Combination of metformin and glucose deprivation enhanced the inhibition of HCC cell migration and invasion

In a previous work, we demonstrated that AMPK is the central kinase controlling apoptotic activation in HCC cells undergoing glucose starvation. In fact, AMPK activation after glucose deprivation is rapid and marked and its silencing completely blocks the augment in the apoptotic population induced by glucose deprivation^15^. Nevertheless, there is no data available regarding the putative effect of this activation on cell migration. Metformin is a well-known antiproliferative and cytotoxic drug, either alone or in combination with another antitumor treatment in diverse cells^26–28^. In HCC derived cells metformin (5 to 10 mM) leads to cell cycle arrest^4,23,29^, and apoptosis^30^. Therefore, we studied if any additive or synergic effect on cell cycle or death, and, more intriguing, on cell migration could be produce by combination of glucose restriction and metformin treatment in HCC cells.

**Figure 4:**
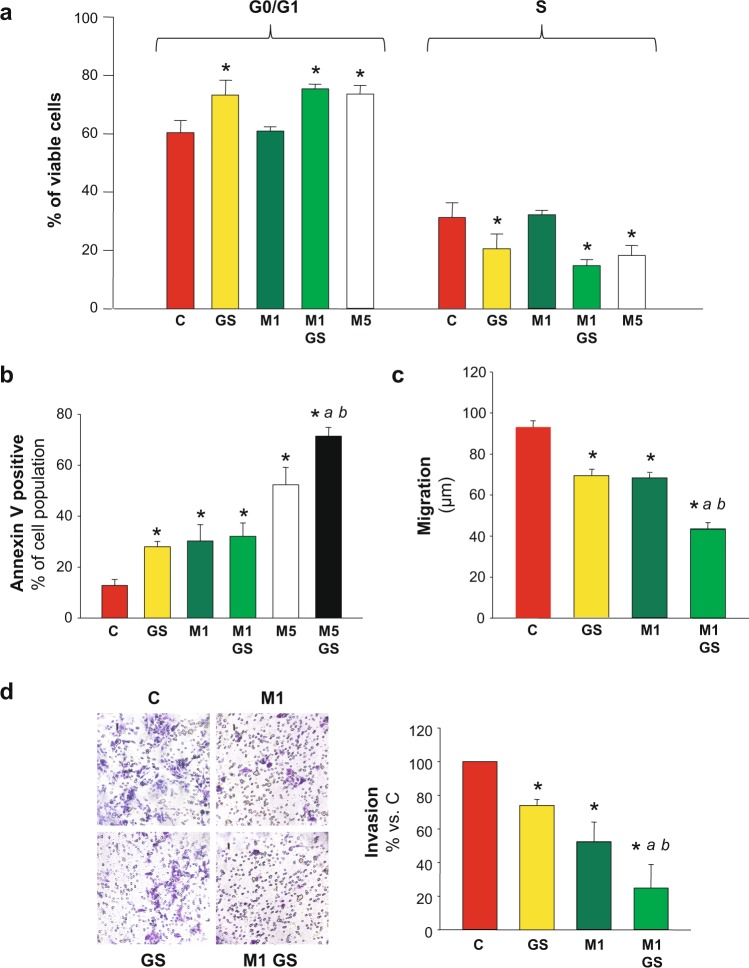
Combination of metformin and glucose deprivation on cell cycle, cell death, migration and invasion. C3A cells were incubated with complete DMEM (C), or glucose starved by incubation in no-glucose DMEM (GS), with or without 1 (M1) or 5 mM (M5) metformin for 48 h. (**a**) Cells were fixed, stained with propidium iodide, and their distribution in cell cycle was analyzed by flow cytometry. Bar charts show the percentage of cells in G0/G1 and S phases. (**b**) Cells were stained for AnnexinV/IP assay and the percentages of apoptotic cells were determined by flow cytometry analysis. Bar represent the total percentages of apopotic cells (Annexin V positive). Values are the mean ± SEM of 3 (cell cycle) or 4 (AnnexinV/IP) independent experiments. (**c**) C3A cells were subjected to scratch wounding (0 h) and incubated with complete DMEM (C), or subjected to glucose starvation (GS), with or without 1 mM metformin (M1) for 24 h. Data were obtained as indicated in Fig. 1b. (**d**) C3A cells were plated in transwell chambers as detailed in Fig. 2 and incubated with complete DMEM (C), or glucose starved (GS), with or without 1 mM metformin (M1) for 48 h, and processed as indicated in Fig. 2. *P < 0.05 *vs*. C. ^*a*^*P* < 0.05 *vs*. GS. ^*b*^*P* < 0.05 *vs*. M5 (**b**) or M1 (**c**,**d**).

Our results showed that 1 mM metformin was not enough to produce cell cycle arrest after 48 h of treatment (Fig. 4a). However, the same treatment conditions significantly increased total apoptosis to almost 30% of the whole cell population (Fig. 4b). In addition, and in accordance with the literature, 5 mM metformin did achieve both significant cell cycle arrest (Fig. 4a and Supplementary Fig. 3), and apoptotic activation (Fig. 4b). When C3A cells were incubated with 5 mM metformin in a glucose starving condition, the percentage of apoptotic cells was significantly enhanced from 52 to 72% (Fig. 4b), whereas the percentage of non-apoptotic viable cells decreased to 18%. For this reason, cell cycle distribution was not analyzed in this group of cells. These results showed that there was a dose dependent effect of metformin on apoptotic activation which was much more prominent in conditions of nutritional stress.

Once these effects on cell cycle and apoptosis were characterized, the effect of combination of 1 mM metformin with glucose deprivation on migration and invasion in HCC cells was analyzed. In C3A cells, glucose starvation doubled the inhibition of migration induced by 1 mM metformin in control conditions: from −27% to −53% (Fig. 4c). Similar results were obtained for HuH-7 cells see Supplementary Fig. 3. In the same direction, 1 mM metformin combined with glucose deprivation prompted a marked inhibition of invasion in C3A cells when compared to metformin alone: from −48% to −75% (Fig. 4d).

Total viability of glucose starved C3A and HuH-7 cells treated with 5 mM metformin for 24 h decreased to almost 65 and 40% of control levels, respectively see Supplementary Fig. 4. Therefore, the effects of treatment combination on cell migration and invasiveness could not be evaluated for 5 mM metformin, because 24 and 48 h assays were required.

### PKA and AMPK signaling in HCC cells exposed to metformin and glucose deprivation

**Figure 5:**
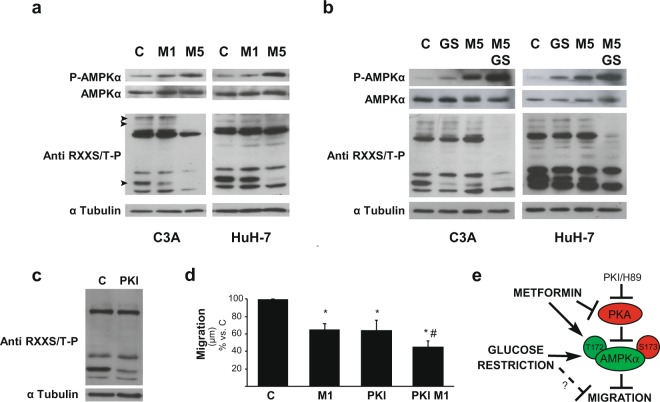
Effect of metformin on PKA/AMPK signaling in glucose fed and glucose fasted cells. (**a**,**b**) HCC cells were incubated with complete DMEM (C), or glucose starved by incubation in no-glucose DMEM (GS), with or without 1 (M1) or 5 mM (M5) metformin for 24 h. Protein levels of P-AMPKα(T172), AMPKα, and phosphorylated PKA substrates were detected. α Tubulin was used as loading control. The bands of PKA substrates which were modified are indicated with arrow heads in the first blot of C3A cells. Full-length blots are available in Supplementary Dataset. Immunoblots show an experiment representative of 3 independent experiments. (**c**) C3A cells were incubated with complete DMEM (C), with or without 10 μM PKI (PKI) for 24 h and phosphorylated PKA substrates were detected in cell lysates. α Tubulin was used as loading control. Selected lanes for each detection are in their original order and correspond to the same gel, and they are shown after cropping, aligning and separating them by white space. Full-length blots are available in Supplementary Dataset. Immunoblots show an experiment representative of 3 independent experiments. (**d**) C3A cells were incubated with complete DMEM (C), in the presence of 1 mM metformin (M1), 10 μM PKI (PKI) or both (PKI M1) for 24 h. Cells were subjected to scratch wounding (0 h) and cell migration was assessed after 24 h, as indicated in Fig. 1d. Bars represent the mean distance migrated by the “wound front” expressed as % of the distance advanced in the control group. Values represent the mean ± SEM from 8–14 fields for an experiment representative of 3 independent experiments. *P < 0.05 vs. C. ^#^*P* < 0.05 vs. M1. (**e**) Proposed model for AMPK activation by PKA inhibitors (metformin, PKI or H89) which entail reduction of HCC cell migration. Glucose starvation also activates AMPK, and under this condition inhibition of migration is reinforced. Mechanisms not yet clarified independent of AMPK activation could also decrease cell migration (dashed line). *P < 0.05 *vs*. C. ^#^*P* < 0.05 *vs*. GS.

Besides its role as an AMPK activator, there is evidence that metformin also acts as a PKA inhibitor in hepatic cells^23^. Therefore, we explored if the increase in the antitumor effects of metformin induced by glucose starvation were associated to changes in the activities of these signaling kinases. As a first approach, we analyzed AMPK and PKA activation, by detecting P-AMPKα(T172) and phosphorylation of PKA substrates in HCC cells cultured in the presence of 1 and 5 mM metformin (Fig. 5a). Metformin treatment increased AMPK activation in a dose dependent manner, and decreased PKA activation at 5 mM. When combined treatment of glucose restriction plus 5 mM metformin was analyzed, we observed that PKA activity was almost undetectable, and activation of AMPK was significantly enhanced (Fig. 5b). This hyper-activation of AMPK paralleled the increase in apoptosis observed by Annexin V/IP assays in the 5 mM metformin plus glucose starvation setting.

It is noteworthy that 1 mM metformin plus glucose starvation did not increase P-AMPKα(T172) levels above those levels elicited by single treatments see Supplementary Fig. 5. In this regard, cell cycle and cell death analysis also showed that combination of 1 mM metformin with glucose withdrawal have no additive effects, what, collectively, indicated that apoptotic death levels correlated with the extent of AMPK activation. However, the same combination of treatments did intensify the inhibition of HCC cell migration/invasion, what could be probably due to additional effects of glucose restriction on these functions which were, at least in part, independent of further AMPK activation.

Subsequently, we focused on the negative regulation of AMPK by PKA in HCC cells. We have previously described that cell death induced by glucose withdrawal in HCC cells is conditioned by PKA inhibition of AMPK^15^ A set of experiments was performed to evaluate migration in the presence of the specifically designed peptide inhibitor of PKA (PKI) (Fig. 5c), 1 mM metformin, or both. Results showed that PKA participated in regulating migration in HCC cells and demonstrated additive effects of PKA inhibition and 1 mM metformin in reducing HCC cell migration (Fig. 5d). Similar data were obtained by using H89 plus 1 mM metformin (data nor shown). We pondered if this axis also affected glucose starvation effects on cell migration. Therefore, we evaluated the effect of the PKA inhibitor H89 on the migratory capacity of control and glucose starved cells. In line with our previous results, inhibition of PKA by H89 reinforced the reduction in the efficiency of C3A cell migration observed during glucose starvation see Supplementary Fig. 6b, and this was associated to the fact that H89 increased AMPK activation in glucose-fasted cells see Supplementary Fig. 6a. Similar results were obtained for HuH-7 cells (data not shown).

All together, these findings supported the hypothesis that PKA counteracted AMPK activation and that higher levels of AMPK activation were achieved in HCC cells when PKA was inhibited by metformin (Fig. 5e).

### Contribution of AMPKα Ser173 site to cell migration

**Figure 6:**
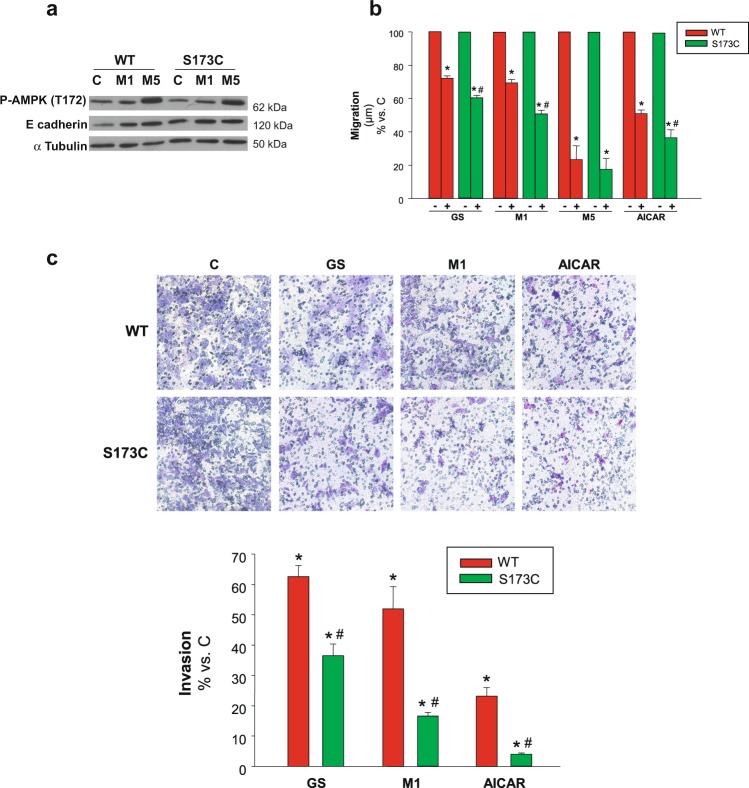
Contribution of Ser173 regulatory site of AMPKα to cell migration/invasion. (**a**) C3A cells stably expressing either AMPKα(S173C) (S173C) or wild type AMPKα(S173) (WT) were incubated with complete DMEM alone (C) or in the presence of 1 mM (M1) or 5 mM (M5) metformin for 24 h. P-AMPKα(T172) and E-cadherin protein levels were detected in cell lysates. α Tubulin was used as loading control. Selected lanes for each detection are in their original order and correspond to the same gel, and they are shown after cropping, aligning and separating them by white space. Full-length blots are available in Supplementary Dataset. Immunoblots show an experiment representative of 3 independent experiments (**b**) C3A cells stably expressing either AMPKα(S173C) (S173C) or wild type AMPKα(S173) (WT) were subjected to scratch wounding (0 h) and incubated in complete DMEM alone (C), or in the presence of 1 mM (M1) or 5 mM metformin (M5), or 1 mM AICAR (AICAR), or glucose starved by incubation in no-glucose DMEM (GS) for 24 h. Data were obtained as indicated in Fig. 1d, and values are expressed as percentages of C. (**c**) S173C and WT cells were plated in transwell chambers as detailed in Fig. 2 and incubated with complete DMEM alone (C), or in the presence of 1 mM metformin (M1), or 1 mM AICAR (AICAR), or subjected to glucose starvation (GS) for 48 h, and processed as described in Fig. 2. Values represent the mean ± SEM of 3 experiments. *P < 0.05 *vs*. C. ^*#*^*P* < 0.05 *vs*. WT.

We demonstrated that the simultaneous treatment of HCC cells with 5 mM metformin and glucose restriction increased AMPK activation, which was concomitant with a decrease of PKA activation. In this connection, a study focused on the response to glucagon in liver cells, showed that metformin inhibits PKA and, concurrently, the specific phosphorylation of AMPKα(S173) by PKA^23^. Our own previous results in HCC cells pointed that mutation of AMPKα(S173) to the non phosphorylatable form AMPKα(S173C) enhanced AMPK activation after glucose deprivation and significantly increased apoptotic death^15^. In the present work, we corroborated that AMPK activation occurred in WT and S173C mutant cells treated with 1 or 5 mM metformin. However, when subjected to 1 mM metformin the mutant clone showed greater induction of T172 phosphorylation. Concomitantly, both in basal and 1mM-metformin conditions, the levels of the epithelial marker E-cadherin were higher in the S173C mutant (Fig. 6a). This indicated that the lack of phosphorylation of S173 (by PKA) favored AMPK activation and hence increased epithelial differentiation. Taking together these results, we hypothesized that the phosphorylation state of this site could modulate AMPK signaling in HCC cells migratory function. To address this hypothesis, wound healing and transwell migration assays were performed in HCC cells harboring non phosphorylatable mutation of the S173 phosphorylation site. In every AMPK activating conditions assessed, migration of AMPKα(S173C) cells was significantly decreased when compared to AMPKαWT cells (Fig. 6b). Besides, when cells were incubated with the concentration of metformin that inhibited PKA (5 mM) the mutation became ineffective (Fig. 6b), which was in accordance with the similar levels of E-cadherin (and activated AMPK) reached in mutant and wild type cells treated with this concentration of metformin (Fig. 6a).

Comparable results were obtained in invasion assays, where, after glucose withdrawal, or incubation with metformin or AICAR, cell invasiveness was significantly decreased in AMPKα(S173C) compared to AMPKαWT cells. As a sake of example, inhibition of invasion increased from 48 to 83% in WT and mutant cells incubated with 1 mM metformin, respectively (Fig. 6c). Summarizing, these results demonstrated that cells harboring an AMPK mutant form that is not inhibitable by PKA had diminished migration and invasion abilities.

## Discussion

EMT is a continuous matter of study in liver cancer due to the presence of fibrosis in the underlying liver disease which, coupled with other special features of the hepatocytes, point to EMT regulation as a target for the control of HCC development^31^. It is well known that AMPK signals epithelial differentiation in primary cultured hepatocytes. Moreover, activation of AMPK by metformin significantly increases epithelial cell polarity in normal hepatocytes^32^. However, the putative AMPK role in the regulation of cell migration process during HCC progression remains unexplored. Here, we aimed to study the effect of AMPK activation on migration and invasion in HCC derived cells and, specifically, when they were treated with the emergent anticarcinogen metformin.

Metformin biological mechanisms and their impacts seem very diverse, and further basic and clinical studies are necessary to understand the complete picture of its antitumor actions^32,33^. In diabetes treatment, metformin has many benefits and a safety dosage, and it became the most useful oral hypoglycemiant^34^. Interestingly, valuable metformin results in patients supporting its possible role as anticarcinogen come from the reduction of cancer frequencies in diabetic patients^35^. Moreover, HCC is prevented in metformin treated patients^36,37^, which is more significant considering that diabetes is a risk factor for HCC^2^. Metformin concentrates in the liver due to the enrichment of the transport protein OCT1 in hepatic cells, which also transports cations as sorafenib^38^. Taken together, this background makes HCC a unique candidate for metformin studies.

First, we showed that a concentration of metformin that causes still meager or no effect on cell cycle and death, does induce significant inhibition of cell migration and invasion in two HCC derived cell lines with different migratory efficiencies. Additionally, either the AMP analog AICAR or glucose starvation induced similar reduction of migration. In turn, we found that metformin inhibitory effects on migration and invasion were abrogated in AMPKα silenced cells. Thus, these results supported that, by activating AMPK, metformin could be an effective antimigratory agent in HCC cells. In addition, activation of AMPK by metformin increased cell differentiation and increased E-cadherin while decreasing vimentin expression in HCC cells. These findings suggested that metformin induced inhibition of cell migration/invasion in HCC cells was due, at least in part, to EMT reversal. Similar data were found in studies performed in diverse cancer cells, from melanoma to breast cancer, which show that treatment with metformin or its functional analogue berberine result in significant diminution of cell migration and invasion via AMPK activation and EMT reduction^39–42^.

Second, we demonstrated that combination of metformin with glucose starvation leads to an enhanced inhibition of cell migration and invasion in both HepG2/C3A and HuH-7 cells. In previous studies, we characterized the role of AMPK signaling in the response of hepatic cancer cells to glucose withdrawal and demonstrated the rapid AMPK activation during glucose restriction and the resultant induction of both G0/G1 cell arrest and apoptotic death^15^. Now we showed that glucose restriction also acts as an antimigratory agent, per se. Furthermore, when cells were exposed to metformin during glucose deprivation, the antitumor effects were increased. The impact of glucose restriction on the migratory ability of HCC cells had not been previously characterized. Metabolic stress during tumor formation activates AMPK, what is proposed to act as an initial prosurvival signal^43^. Nevertheless, most findings in different tumor cells support antitumor effects of AMPK activation by energy stress (starvation or 2-deoxyglucose treatment), including novel putative targets and inhibition of migration and invasion^14,44^. Further studies will be necessary to determine if one or more of these specific targets involved in migration/invasion are responsible of metformin triggered AMPK signaling and hence inhibit migration and invasion in HCC cells. However, our results support that, at least in part, an intact AMPK-p53 axis is required to signal these inhibitions. In fact, and in accordance with previous evidence observed in normal liver under energetic stress which suggest p53 stabilization by AMPK phosphorylation^24^, we demonstrated that AMPK activation through metformin is associated with an increase of p53 in HCC cells, and that cells lack of p53 show no inhibition of migration/invasion by metformin. Similar dependence on p53 was suggested in metformin induced apoptosis in breast cancer cells^45^. Given that p53 per se can regulate EMT and migration/invasion and that p53 also interplays with other direct targets of AMPK implicated in migration and invasion processes in diverse cancer cells, additional approaches of analysis are required to elucidate the role of AMPK-p53 and the downstream actors involved in the regulation of migration and invasion in HCC cells.

More importantly, here, we demonstrated that AMPK activation either by energy stress or by the pharmacological action of metformin led to the inhibition of migration/invasion of HCC cells, which was related with metformin concentrations and, thus, with the extent of AMPK activation. Moreover, glucose deprivation turned HCC cells migratory and invasive properties extremely sensitive to metformin treatment. In addition, metformin treatment of glucose starved cells also increased apoptotic death, what was even more pronounced at a metformin concentration that inhibited PKA activity. In fact, under glucose deprivation, the drastic inhibition of PKA entailed by 5 mM metformin was associated to a major increase of AMPK activation.

Given that metformin treatment reduces PKA mediated phosphorylation of AMPKα(Ser173) and hence facilitates AMPK activation in hepatic cells^23^, we explored the possible contribution of S173 residue of AMPKα to the inhibition of migration/invasion elicited by AMPK activation. We found that cells harboring the non-phosphorylatable S173C mutation presented decreased migration and invasion efficiencies when subjected to AMPK activating conditions. However, in the presence of 5 mM metformin, a concentration which inhibited PKA, migration was decreased to a minimum irrespective of the mutation of S173. In this sense, we concluded, as a third main point, that S173 phosphorylation constrains AMPK activation and affects migratory functions in such a way that conditions which prevent its phosphorylation by PKA enhance AMPK antimigratory effects.

Results in cultured tumor cells, subjected to their usual *in vitro* conditions of extremely high supply of glucose and growth factors, are observed with mM instead of μM metformin concentrations, which are the plasmatic levels in patients^46^. However, those concentrations were unlikely to be supra pharmacological because metformin concentrates in tissues several folds over blood levels and bioavailability in the liver reaches 8 mM^47^. Moreover, other antitumor effects via AMPK, analyzed firstly in cultured HCC cells, were also demonstrated in HCC cell-xenotransplantated mice treated with metformin at pharmacological doses^4,29^, this supporting further translational hypotheses and clinical studies with metformin in HCC patients from *in vitro* evidence. On the other hand, many results encourage the use of caloric restriction or periodic fasting for cancer treatment and prevention, and diverse clinical trials are now in course^48^. Changes in signaling kinases are proposed to mediate these dietary antitumor effects, and AMPK is one of the candidates which was studied in rodents but not yet in patients. In fact, caloric restriction decreases tumor development in a breast cancer model in mice and this is associated with AMPK activation in tumor, mammary gland and liver^49^.

Collectively, we presented evidence regarding novel mechanisms responsible for regulating the migratory and invasive abilities of HCC cells. We focused on conditions that enhance AMPK signaling and result in greater inhibition of cell migration and invasion, as AMPK sensitivity to inhibition by PKA. Due to HCC special features, which comprise accumulation of mutations during chronic inflammation and rapid intrahepatic metastasis, it is a molecular/genetically heterogeneous cancer lacking in effective treatment^50,51^. In this regard, metabolic or dietary interventions combined with drugs targeted to signaling kinases as metformin, accompanied of early detection can be a promising therapy for limiting the rapid metastatic course of this disease.

## Methods

### Cell culture

The hepatocarcinoma cells C3A (HepG2/C3A, a clonal derivative of HepG2, ATCC, Manassas, VA), HuH-7 (JCRB Cell Bank, Tokyo, Japan), and Hep3B (Hep 3B2.1–7, ATCC) were grown with 4.5 g/L glucose DMEM or, in the case of glucose restriction experiments, with no-glucose DMEM (Gibco, Thermo Fisher Scientific, Waltham, MA), supplemented with 10% FBS and antibiotics. When indicated, 1,1-Dimethylbiguanide Hydrochloride (1–20 mM, Metformin) (Santa Cruz Biotechnology Inc., Santa Cruz, CA), the AMPK activator 5-Aminoimidazole-4-carboxamide ribonucleotide (1 mM, AICAR) (Cell Signaling Technology, Danvers, MA) or the PKA inhibitors N-[2-(p-Bromocinnamylamino)ethyl]-5-isoquinolinesulfonamide dihydrochloride (5 µM, H89) (Santa Cruz Biotechnology Inc.) or Protein Kinase A inhibitor fragment 14–22, myristoylated (10 µM, PKI) (Sigma Chemical Co., St Louis, MO) were added.

### Reduction of AMPKα expression

For reducing AMPKα1 protein expression in HCC cells, 21 nucleotide duplexes targeting two specific AMPKα1 sequences and scrambled control were designed and synthesized by Ambion SilencerTMsiRNA (Thermo Fisher Scientific) and cells were transfected, as we previously described^15^. The target sequences corresponded to AMPKα1 1842–1864: AACATTTCTGCATATTAGGCTCCTGTCTC and 2659–2681: AAGAGCTGAGTTGCATATACTCCTGTCTC, as we previously used^15^. Experiments were performed 24–48 h after transfection, and the specific decrease in AMPKα expression for both duplexes was confirmed by immunoblotting. Reductions of 70–80% of protein expression were achieved in both cell lines.

### Generation of stable cell lines

pCDNA3 plasmid harboring Myc-AMPKα1(WT) and Myc-AMPKα1(S173C)^52,53^, kindly given by Dr. Dietbert Neumann (Maastricht University), were used to generate populations of C3A cells stably expressing WT and mutated forms of the AMPKα1 verified by detection of cMyc and AMPKα, as we previously described^15^. The stable clones obtained were grown in a medium containing 200 μg/ml Geneticin (Invitrogen, Thermo Fisher Scientific), in conditions otherwise similar to that of the parental cells.

### MTT assay

Cells were cultured in 96-well microplates and methylthiazolyldiphenyl-tetrazolium bromide (MTT, Sigma Chemical Co., St Louis, MO) was added at the indicated time, as we previously described^15^. After 2 h, the metabolite produced from viable cells was dissolved in DMSO and absorbance was detected at 540 nm in a microplate reader (Beckman Coulter LD400). Results were expressed as percentage of absorbance in control cells.

### BrdU incorporation assay

Cells were plated onto coverslips, subjected to the indicated treatments and then BrdU (10 mM) was added and cells were incubated for additional 3 h. After fixed in 4% paraformaldehyde and permeabilized with Triton X-100, cells were washed three times with PBS for 5 min and DNA was denaturalized by incubating 10 seconds in 50 mM NaOH. After treatment with a blocking solution, the cells were incubated with anti-BrdU antibody (B8434 Sigma Chemical Co.), washed and incubated with secondary antibody, washed once again and incubated with 4′,6-diamidino-2-phenylindole (DAPI, Molecular Probes) for nuclei staining. The cells were then washed and mounted for observation under a fluorescence microscope (Nikon TE200). Independent areas were captured and BrdU-positive nuclei were counted and expressed as percentage (a total of 500 nuclei on average were counted).

### Annexin V/propidium iodide assay

Cells were prepared for Annexin V assay (BD Biosciences, San José, CA), as we previously described^15^. In brief, after gently homogenization in the culture medium and harvest, 100,000 cells were carefully re-suspended and externalization of phosphatidylserine and cell death was assessed by staining with Annexin V-FITC and PI, respectively, coupled to flow cytometric analysis (Cell Sorter BD FACSAria II, BD Biosciences), following the manufacturers’ instructions.

### Cell cycle analysis

Cell distribution in the cell cycle was analyzed by determining the cellular DNA content by flow cytometry, as we previously described^15^. Briefly, 1 × 10^6^ cells were fixed with cold 70% ethanol, washed with PBS and stained with 50 μg/ml propidium iodide (Sigma Chemical Co.) in a buffer solution (0.1% sodium citrate, 0.02 mg/ml RNAse, and 0.3% NP-40). Results were analyzed using WinMDi and Cylchred softwares.

### Immunofluorescence confocal microscopy

Cells were grown on glass coverslips and, at the end of experiments, fixed and treated, as we previously described^25,54^. Briefly, fixed cells were permeabilized and blocked with 0.3% Triton X-100-1% albumin. Then they were incubated for F-actin staining with Alexa 560-conjugated phalloidin (Molecular Probes, Eugene, OR), washed, incubated for nuclei staining with 4′,6-diamidino-2-phenylindole (DAPI, Molecular Probes), and mounted with ProLong (Molecular Probes). Fluorescence was detected by using confocal microscopy (Nikon C1SiR with inverted microscope Nikon TE200). Serial optical sections in the z-axis were collected, and projections were obtained using ImageJ tools. Canalicular structures were identified and quantified, as we previously described^25^.

### Wound healing assay

Cell monolayers were subjected to wound healing assay to measure collective cell migration, as we previously described^55^, with slight modifications. In brief, cells seeded at 3 × 10^6^ (C3A) or 1,5 × 10^6^ (HuH-7) in DMEM were cultured in 6-well plates (or 24 well-plates in the case of PKI experiments). After 24 h, cells were wounded by dragging a 200-μl pipette tip through the monolayer, washed and subjected to their respective treatments. Microscopic images (Zeiss Axiovert 25) of wounds in the same field were captured when the wound was made (0 h) and 24 h after wounding. The length (μm) advanced by the cells of the wound front was assessed by using ImageJ software. For time lapse studies, cells were cultured in 35 mm dishes and wounds were dragged with a 20-μl pipette tip. The cells were otherwise treated as described above and sequential pictures from the same field over a plate thermostated at 37 °C were automatically captured every 30 min for the last 3 h within 24 h of treatment. The advancing of a single cell in the wound front was assessed (a line segment from the initial to the final centriole position) by using ImageJ software.

### Invasion assay

Invasion assay was performed using a transwell chamber (Biofil, Beijing, China) with 8 μm pore size polyester membrane filters. The upper side was pre-coated with 250 μg/ml Matrigel (Corning, Corning, NY). Cells were trypsinized and suspended in DMEM containing 1% FBS. Next, 1 × 10^5^ cells were added to the upper chamber, and the lower chamber was filled with complete DMEM medium containing 10% FBS without any modification throughout the experiment. Cells were subjected to their respective treatments for 48 h. After incubating, cells that had invaded the lower chamber were fixed with methanol, stained with 1% Toluidine Blue in 1% borax for 5 min, and counted using an inverted microscope.

### Preparation of cell lysates

Whole cell lysates were prepared, as we previously described^15^. Briefly, cell monolayers were washed, scrapped and harvested by centrifugation. Pellets were incubated for 30 min in RIPA buffer (1% Triton X-100 (v/v), 1% sodium deoxycholate (w/v), 0.1% SDS (w/v), 20 mM Tris, pH 8, 5 mM EDTA, 200 mM NaCl) supplemented with protease (1 mM PMSF, 10 μg/mL leupeptin (Sigma Chemical Co.)) and phosphatase (10 mM NaF, 2 mM Na_3_VO_4_, 100 nM calyculin A (Sigma Chemical Co.)) inhibitors, and then sonicated. Protein concentration was measured according to Lowry *et al*.^56^.

### Immunoblotting

Total proteins were separated by electrophoresis on SDS-polyacrylamide gels^57^, and transferred to polyvinylidene difluoride membranes (Perkin Elmer Life Sciences, Boston, MA, USA). Membranes were blocked with 5% non-fat milk/0.3% Tween/PBS. In the case of phospho-ACC and ACC detections, electrophoreses were performed in 6–10% gels for probing in the same samples those greater proteins (phospho-ACC, ACC) with regular size proteins (AMPKα, p53, etc.), and the upper halves of the gels were transfer for 3 h to nitrocellulose membranes and blocked with 3% albumin 0.3%Tween/TBS. All the membranes were washed and incubated overnight with specific primary antibodies [α tubulin (T-5168 Sigma Chemical Co.); p-ACCa (Ser 78/Ser 80)-R, ACCa (D5), p53(DO-1) (sc-30447-R, sc-137104, sc-126 Santa Cruz Biotechnology); phospho-(Ser/Thr) PKA Substrate, AMPKα, Phospho-AMPKα (Thr172), (9621S, 2532, 2535 Cell Signaling Technology); vimentin (M0725 Dako, Denmark); or E-cadherin (610182 BD Biosciences)]. Membranes were probed with the appropriate secondary antibody. Bands were detected by chemiluminescence (Amersham Pharmacia Biotech, Piscataway, NJ).

### Statistical analysis

Data were expressed as mean ± SEM. Student *t* test was used for comparison between groups. *P* < 0.05 was considered statistically significant.

## Supplementary information

Supplementary figures

Dataset 1

## References

[1] Uchino K (2011). Hepatocellular carcinoma with extrahepatic metastasis: clinical features and prognostic factors. Cancer.

[2] Marquardt JU, Andersen JB, Thorgeirsson SS (2015). Functional and genetic deconstruction of the cellular origin in liver cancer. Nat. Rev. Cancer.

[3] Llovet JM, Villanueva A, Lachenmayer A, Finn RS (2015). Advances in targeted therapies for hepatocellular carcinoma in the genomic era. Nat. Rev. Clin. Oncol..

[4] Cheng J (2014). AMP-activated protein kinase suppresses the *in vitro* and *in vivo* proliferation of hepatocellular carcinoma. PloS One.

[5] Yu R (2014). Berberine-induced apoptotic and autophagic death of HepG2 cells requires AMPK activation. Cancer Cell Int..

[6] Lee CW (2012). AMPK promotes p53 acetylation via phosphorylation and inactivation of SIRT1 in liver cancer cells. Cancer Res..

[7] Vara D (2011). Anti-tumoral action of cannabinoids on hepatocellular carcinoma: role of AMPK-dependent activation of autophagy. Cell Death Differ..

[8] Hardie DG, Ross FA, Hawley SA (2012). AMPK: a nutrient and energy sensor that maintains energy homeostasis. Nat. Rev. Mol. Cell. Biol..

[9] Jones RG (2005). AMP-activated protein kinase induces a p53-dependent metabolic checkpoint. Mol. Cell.

[10] Liang J, Mills GB (2013). AMPK: a contextual oncogene or tumor suppressor?. Cancer Res..

[11] Hardie DG (2015). Molecular Pathways: Is AMPK a Friend or a Foe in Cancer?. Clin. Cancer Res..

[12] Nagalingam A, Arbiser JL, Bonner MY, Saxena NK, Sharma D (2012). Honokiol activates AMP-activated protein kinase in breast cancer cells via an LKB1-dependent pathway and inhibits breast carcinogenesis. Breast Cancer Res..

[13] Chou CC (2014). AMPK reverses the mesenchymal phenotype of cancer cells by targeting the Akt–MDM2–Foxo3a signaling axis. Cancer Res..

[14] Yan Y (2015). Augmented AMPK activity inhibits cell migration by phosphorylating the novel substrate Pdlim5. Nature Commun..

[15] Ferretti AC (2016). AMPK and PKA interaction in the regulation of survival of liver cancer cells subjected to glucose starvation. Oncotarget.

[16] Djouder, N. *et al*. PKA phosphorylates and inactivates AMPKalpha to promote efficient lipolysis. *EMBO J*. **29**, 469-481 (2010). 10.1038/emboj.2009.339 PMC2824464 19942859

[17] Hurley RL (2006). Regulation of AMP-activated protein kinase by multisite phosphorylation in response to agents that elevate cellular cAMP. J. Biol. Chem..

[18] Mankouri J (2010). Enhanced hepatitis C virus genome replication and lipid accumulation mediated by inhibition of AMP-activated protein kinase. Proc. Natl. Acad. Sci. USA.

[19] Sahra IB, Le Marchand-Brustel Y, Tanti JF, Bost F (2010). Metformin in cancer therapy: a new perspective for an old antidiabetic drug?. Mol. Cancer Ther..

[20] Zhou G (2001). Role of AMP-activated protein kinase in mechanism of metformin action. J. Clin. Invest..

[21] Xie Z, Dong Y, Scholz R, Neumann D, Zou MH (2008). Phosphorylation of LKB1 at serine 428 by protein kinase C-ζ is required for metformin-enhanced activation of the AMP-activated protein kinase in endothelial cells. Circulation.

[22] Xie Z (2009). Identification of the serine 307 of LKB1 as a novel phosphorylation site essential for its nucleocytoplasmic transport and endothelial cell angiogénesis. Mol. Cell. Biol..

[23] Aw DKL, Sinha RA, Xie SY, Yen PM (2014). Differential AMPK phosphorylation by glucagon and metformin regulates insulin signaling in human hepatic cells. Biochem. Biophys. Res. Commun..

[24] Prokesch, A. *et al*. Liver p53 is stabilized upon starvation and required for amino acid catabolism and gluconeogenesis. *FASEB J.* **31**, 732–742 (2017). 10.1096/fj.201600845R PMC5240663 27811061

[25] Mattaloni SM (2012). AKAP350 Is involved in the development of apical “canalicular” structures in hepatic cells HepG2. J. Cell Physiol..

[26] Ben Sahra IB (2010). Targeting cancer cell metabolism: the combination of metformin and 2-deoxyglucose induces p53-dependent apoptosis in prostate cancer cells. Cancer Res..

[27] Silvestri A (2015). Metformin induces apoptosis and downregulates pyruvate kinase M2 in breast cancer cells only when grown in nutrient-poor conditions. PloS one.

[28] Wang LW (2008). Metformin induces apoptosis of pancreatic cancer cells. World J. Gastroenterol..

[29] Miyoshi H (2014). Effect of the anti-diabetic drug metformin in hepatocellular carcinoma *in vitro* and *in vivo*. Int. J. Oncol..

[30] Bhat M (2017). Metformin requires 4E-BPs to induce apoptosis and repress translation of Mcl-1 in hepatocellular carcinoma cells. Oncotarget.

[31] Giannelli G, Koudelkova P, Dituri F, Mikulits W (2016). Role of epithelial to mesenchymal transition in hepatocellular carcinoma. J. Hepatol..

[32] Fu D, Wakabayashi Y, Ido Y, Lippincott-Schwartz JI, Arias M (2010). Regulation of bile canalicular network formation and maintenance by AMP-activated protein kinase and LKB1. J. Cell Sci..

[33] Foretz M, Guigas B, Bertrand L, Pollak M, Viollet B (2014). Metformin: from mechanisms of action to therapies. Cell Metab..

[34] Inzucchi SE, Lipska KJ, Mayo H, Bailey CJ, McGuire DK (2014). Metformin in patients with type 2 diabetes and kidney disease: a systematic review. JAMA.

[35] Decensi A (2010). Metformin and cancer risk in diabetic patients: a systematic review and meta-analysis. Cancer Prev. Res. (Phila).

[36] Hassan MM (2010). Association of diabetes duration and diabetes treatment with the risk of hepatocellular carcinoma. Cancer.

[37] Chen HP (2013). Metformin decreases hepatocellular carcinoma risk in a dose-dependent manner: population-based and *in vitro* studies. Gut.

[38] Hyrsova L, Smutny T, Trejtnar F, Pavek P (2016). Expression of organic cation transporter 1 (OCT1): unique patterns of indirect regulation by nuclear receptors and hepatospecific gene regulation. Drug Metab. Rev..

[39] Cerezo M (2013). Metformin blocks melanoma invasion and metastasis development in AMPK/p53-dependent manner. Mol. Cancer Ther..

[40] Qu C (2014). Metformin reverses multidrug resistance and epitelial-mesenchymal transition (EMT) via activating AMP-activated protein kinase (AMPK) in human breast cancer cells. Mol. Cell. Biochem..

[41] Kim HS (2012). Berberine-induced AMPK activation inhibits the metastatic potential of melanoma cells via reduction of ERK activity and COX-2 protein expression. Biochem. Pharmacol..

[42] Park JJ (2012). Berberine inhibits human colon cancer cell migration via AMP-activated protein kinase-mediated downregulation of integrin β1 signaling. Biochem. Biophys. Res. Commun..

[43] Jeon SM, Chandel NS, Hay N (2012). AMPK regulates NADPH homeostasis to promote tumour cell survival during energy stress. Nature.

[44] Schaffe BE (2015). Identification of AMPK phosphorylation sites reveals a network of proteins involved in cell invasion and facilitates large-scale substrate prediction. Cell Metab..

[45] Li P, Zhao M, Parris AB, Feng X, Yang X (2015). p53 is required for metformin-induced growth inhibition, senescence and apoptosis in breast cancer cells. Biochem. Biophys. Res. Commun..

[46] Lalau JD, Lemaire-Hurtel AS, Lacroix C (2011). Establishment of a database of metformin plasma concentrations and erythrocyte levels in normal and emergency situations. Clin. Drug Invest..

[47] Martin-Castillo B, Vazquez-Martin A, Oliveras-Ferraros C, Menendez JA (2010). Metformin and cancer: doses, mechanisms and the dandelion and hormetic phenomena. Cell Cycle.

[48] Buono R, Longo VD (2018). Starvation, Stress Resistance, and Cancer. Trends Endocrinol. Metab..

[49] Jiang W, Zhu Z, Thompson HJ (2008). Dietary energy restriction modulates the activity of AMP-activated protein kinase, Akt, and mammalian target of rapamycin in mammary carcinomas, mammary gland, and liver. Cancer Res..

[50] Schulze K, Nault JC, Villanueva A (2016). Genetic profiling of hepatocellular carcinoma using next-generation sequencing. J. Hepatol..

[51] Li L, Wang H (2016). Heterogeneity of liver cancer and personalized therapy. Cancer Lett..

[52] Neumann D, Woods A, Carling D, Wallimann T, Schlattner U (2003). Mammalian AMP-activated protein kinase: functional heterotrimeric complexes by co-expression of subunits in *Escherichia coli*. Protein Expr. Purif..

[53] Woods A (2003). Identification of phosphorylation sites in AMP activated protein kinase (AMPK) for upstream AMPK kinases and study of their roles by site directed mutagenesis. J. Biol. Chem..

[54] Ferretti AC, Mattaloni SM, Ochoa JE, Larocca MC, Favre C (2012). Protein kinase A signals apoptotic activation in glucose-deprived hepatocytes: participation of reactive oxygen species. Apoptosis.

[55] Tonucci FM (2015). Centrosomal AKAP350 and CIP4 act in concert to define the polarized localization of the centrosome and Golgi in migratory cells. J. Cell Sci..

[56] Lowry OH, Rosebrough NJ, Farr AL, Randall RJ (1951). Protein measurement with the Folin phenol reagent. J. Biol. Chem..

[57] Laemmli UK (1970). Cleavage of structural proteins during the assembly of the head of bacteriophage T4. Nature.

