# Supplementary material for: Metformin and glucose starvation decrease the migratory ability of hepatocellular carcinoma cells: targeting AMPK activation to control migration
Source: Sci Rep. 2019 Feb 26;9:2815. doi: 10.1038/s41598-019-39556-w (PMC6391381; doi:10.1038/s41598-019-39556-w)
Supplement: Supplementary file 1 — Supplementary figures [file 41598_2019_39556_MOESM1_ESM.pdf]

Title: Metformin and glucose starvation decrease the migratory ability of hepatocellular carcinoma cells: targeting AMPK activation to control migration

Authors: Anabela C. Ferretti, Florencia Hidalgo, Facundo M. Tonucci, Evangelina Almada, Alejandro Pariani, María C. Larocca, Cristián Favre

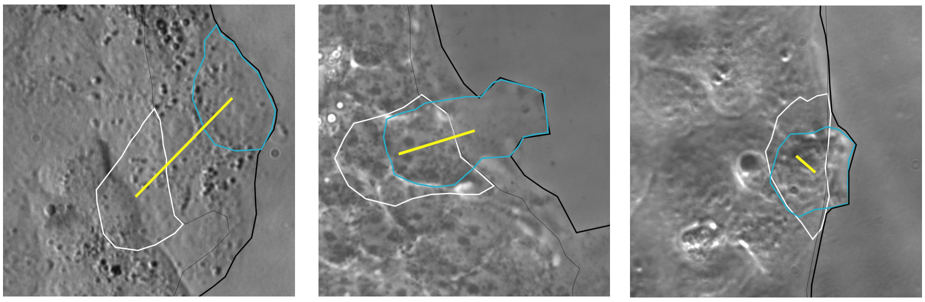

C **M1** **GS**

Supplementary figure 1. Migration of individual cells in the wound front. HuH-7 cells were incubated with complete DMEM (C), with 1 mM metformin (M1), or glucose starved by incubation in no-glucose DMEM (GS) and monolayers were “wounded” as described in Methods. Pictures from the same field were automatically captured every 30 min for 3 h. ImageJ software was used for image analysis. For the sake of figure simplification only the last contrast phase image of the serie is shown and the matching data from the initial image are overlapped. The advancing of a single cell in the wound front (thin and thick black lines indicate initial and final wound front, respectively) was assessed as the line segment (yellow line) from the initial (cell delimited in white) to the final (cell delimited in cian) centriole position. The images illustrate typical individual measurements from three independent experiments. The advancing determined in each experimental group correspond to 17.75, 8.72 and 2.95 μm, respectively

a

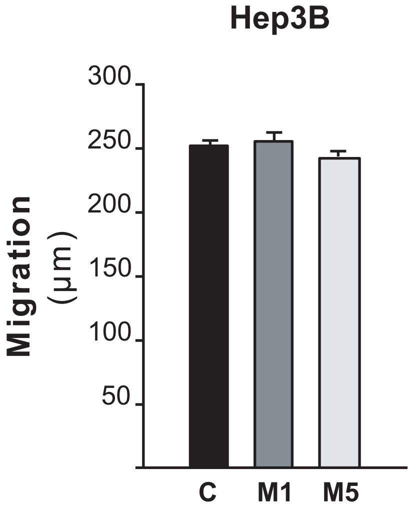

b

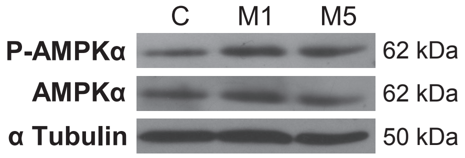

Supplementary figure 2. Migration in metformin treated p53 null HCC cells. Hep3B cells were incubated with complete DMEM (C), plus 1 (M1) or 5 mM (M5) metformin for 24 h. (a) Cells were subjected to scratch wounding (0 h) and cell migration was assessed after 24 h, as indicated in Figure 1d. (b) P-AMPKα(T172) and AMPKα protein levels were detected in cell lysates. α Tubulin was used as loading control. Selected lanes for each detection are in their original order and correspond to the same gel, and they are shown after cropping, aligning and separating them by white space. Full-length blots are available in Supplementary Dataset. Immunoblots show an experiment representative of 3 independent experiments.

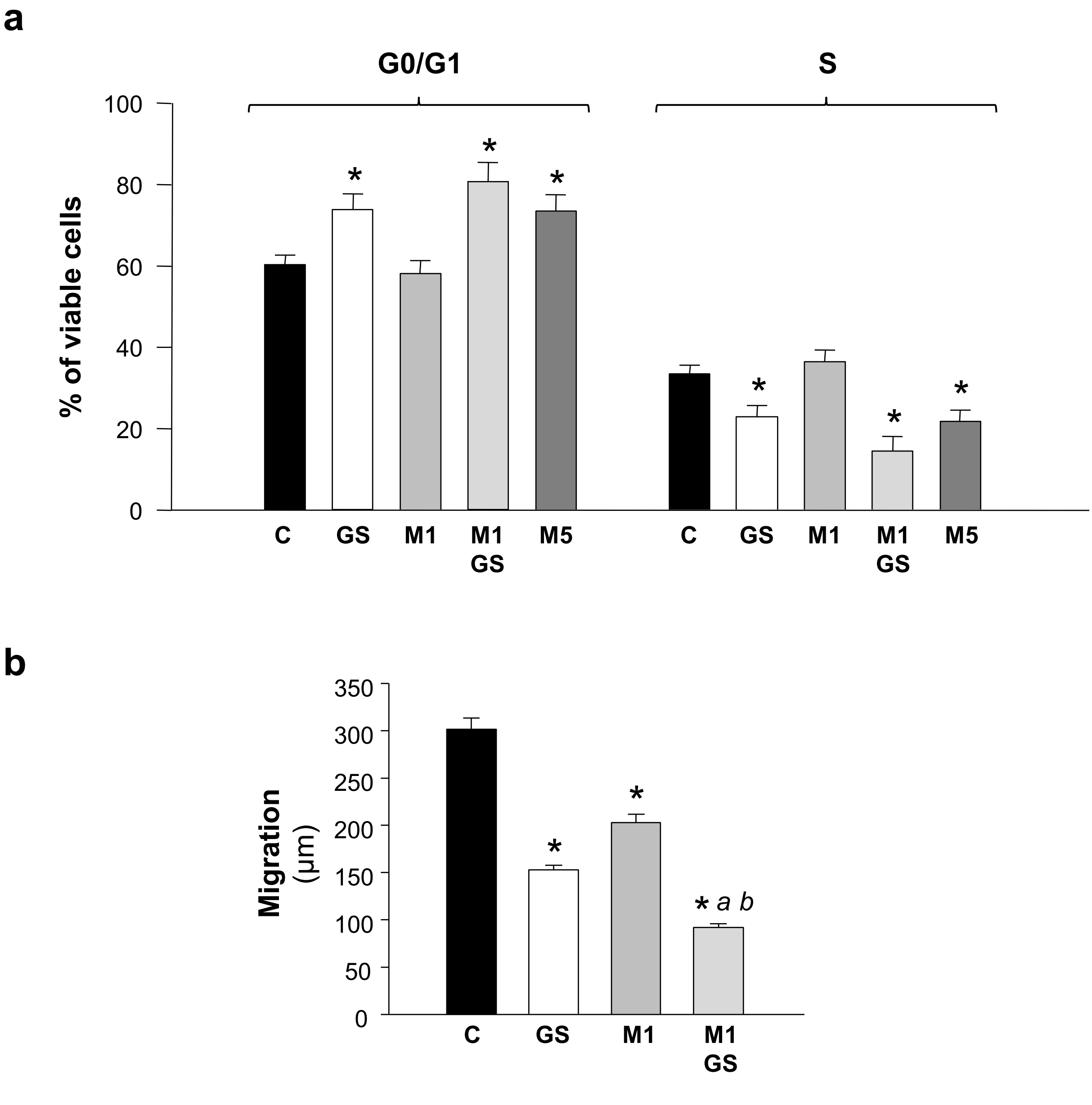

Supplementary figure 3. Effects of metformin and glucose starvation combined treatment on cell cycle and migration in HuH-7 cells. (**a**) HuH-7 cells were incubated with complete DMEM (C), or glucose starved by incubation in no-glucose DMEM (GS), with or without 1 (M1) or 5 mM (M5) metformin for 48 h. Cells were fixed, stained with propidium iodide, and their distribution in cell cycle was analyzed by flow cytometry. Bar charts show the percentage of cells in G0/G1 and S phases. (**b**) Confluent HuH-7 cells were subjected to scratch wounding (0 h) and incubated with complete DMEM (C), or glucose starved by incubation in no-glucose DMEM (GS), with or without 1 mM metformin (M1) for 24 h. Bars represent the mean distance migrated by the "wound front" at 24 h. *P<0.05 vs. C. ^{a}P<0.05 vs. GS. ^{b}P<0.05 vs. M1.

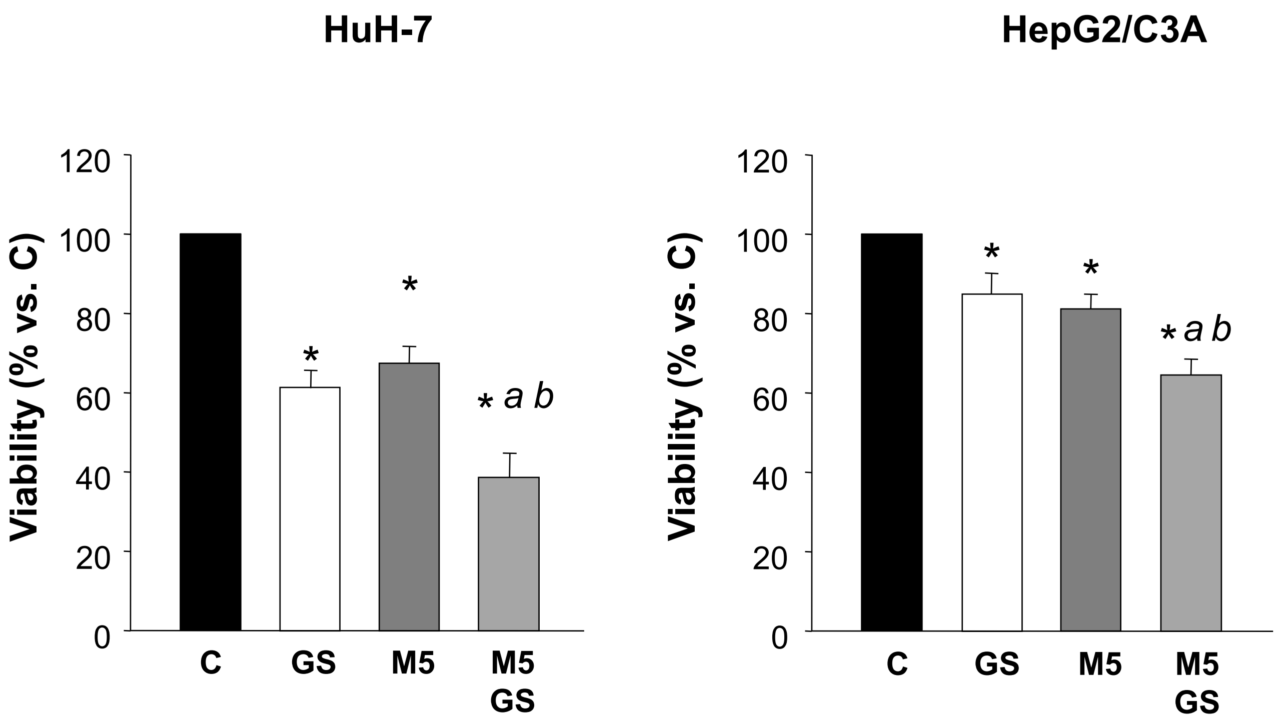

Supplementary figure 4. Survival of HCC cells subjected to 5 mM metformin and glucose starvation for 24 h. HCC cells were incubated with complete DMEM (C), or glucose starved by incubation in no-glucose DMEM (GS), with or without 5 mM (M5) metformin for 24 h, and cell viability was evaluated by MTT assay. Bars represent the mean cell viability expressed as percentage of C ± SEM of 3 independent experiments.*P<0.05 vs. C. ^{a}P<0.05 vs. GS. ^{b}P<0.05 vs. M5.

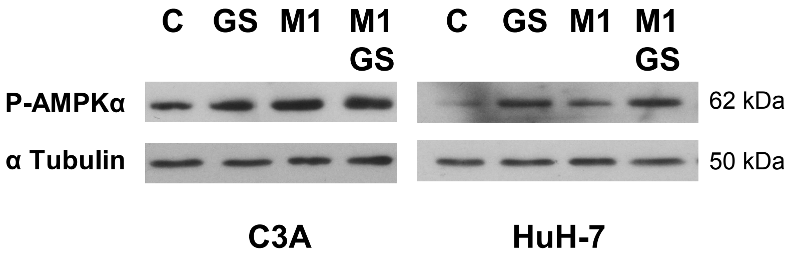

Supplementary figure 5. Effects of 1 mM metformin and glucose starvation on AMPK activation in HCC cells. HCC cells were incubated with complete DMEM (C), or glucose starved by incubation in no-glucose DMEM (GS), with or without 1 mM (M1) metformin for 24 h. Protein levels of P-AMPKα(T172) were detected. α Tubulin was used as loading control. Immunoblots show an experiment representative of 3 independent experiments.

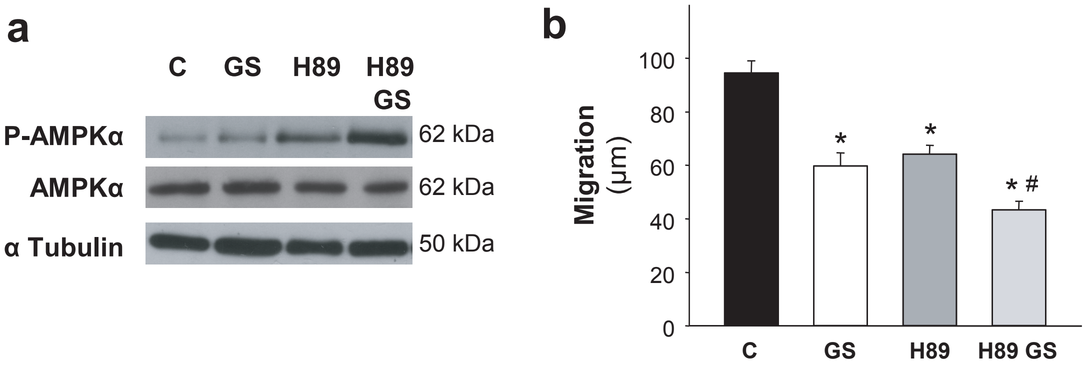

Supplementary figure 6. Migration in glucose starved or H89 treated HCC cells. C3A cells were incubated with complete DMEM (C), or subjected to glucose starvation (GS), with or without 5 µM H89 (H89) for 24 h. (a) P-AMPKα(T172) and AMPKα protein levels were detected in cell lysates. α Tubulin was used as loading control. Selected lanes for each detection are in their original order and correspond to the same gel, and they are shown after cropping, aligning and separating them by white space. Full-length blots are available in Supplementary Dataset. Immunoblots show an experiment representative of 3 independent experiments. (b) Cells were subjected to scratch wounding (0 h) and cell migration was assessed after 24 h, as indicated in Figure 1d. *$P<0.05$ vs. C. #$P<0.05$ vs. GS.
