# Supplementary material for: Metformin and glucose starvation decrease the migratory ability of hepatocellular carcinoma cells: targeting AMPK activation to control migration
Source: Sci Rep. 2019 Feb 26;9:2815. doi: 10.1038/s41598-019-39556-w (PMC6391381; doi:10.1038/s41598-019-39556-w)
Supplement: Supplementary file 2 — Dataset 1 [file 41598_2019_39556_MOESM2_ESM.pdf]

Title: Metformin and glucose starvation decrease the migratory ability of hepatocellular carcinoma cells: targeting AMPK activation to control migration

Authors: Anabela C. Ferretti, Florencia Hidalgo, Facundo M. Tonucci, Evangelina Almada, Alejandro Pariani, María C. Larocca, Cristián Favre

RAW DATA FIG. 2

C3A

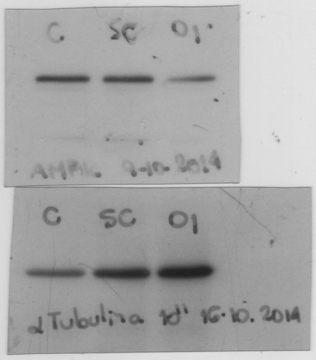

RAW DATA FIG. 2

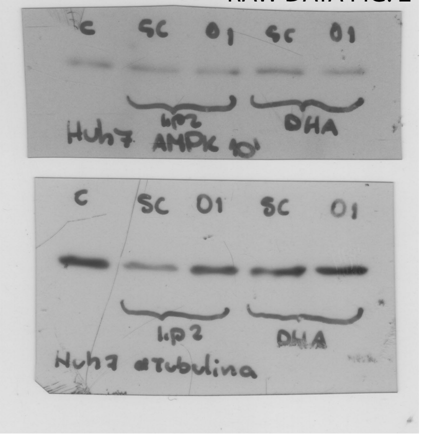

RAW DATA FIG. 3

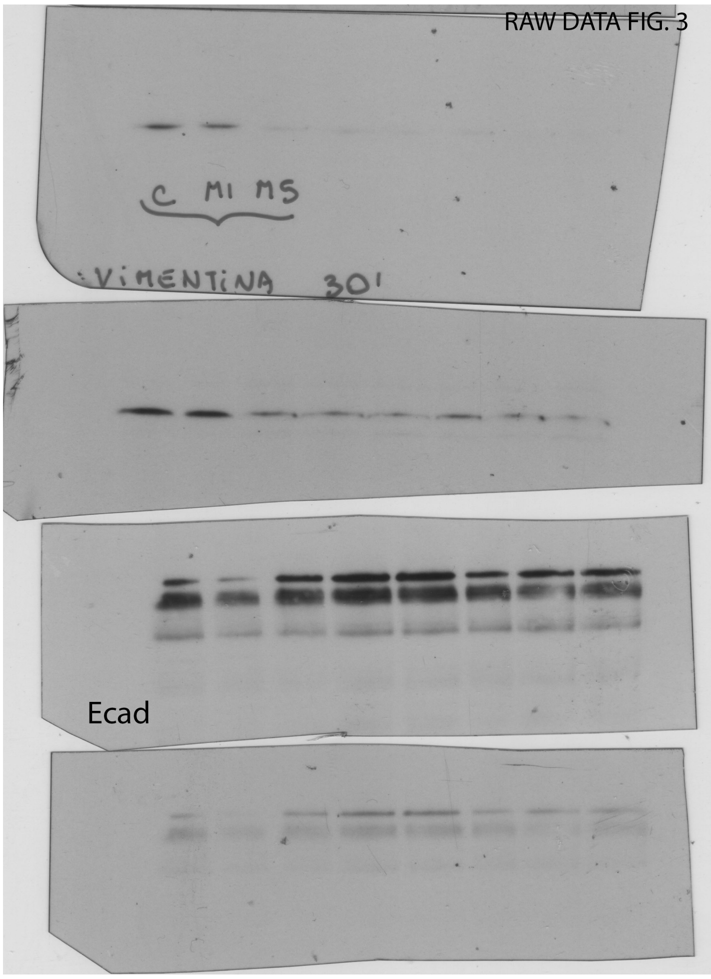

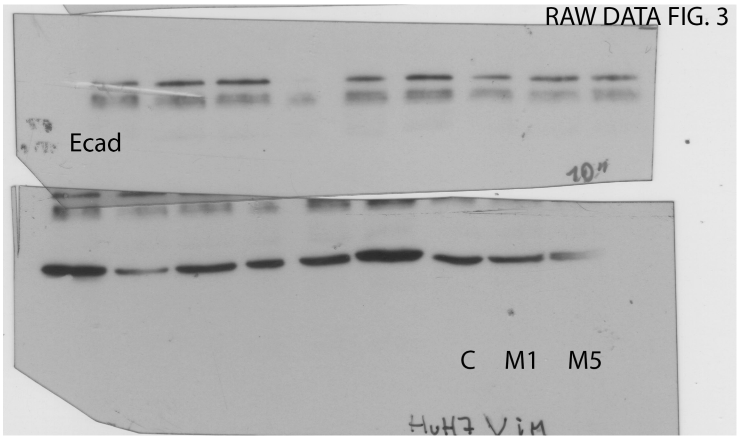
RAW DATA FIG. 3
Ecad
C M1 M5

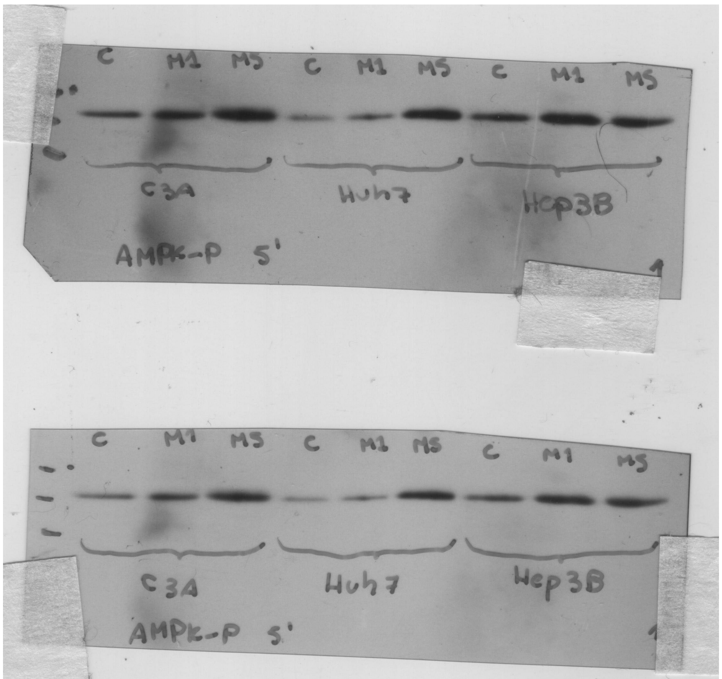

RAW DATA FIG. 5

RAW DATA FIG. 5

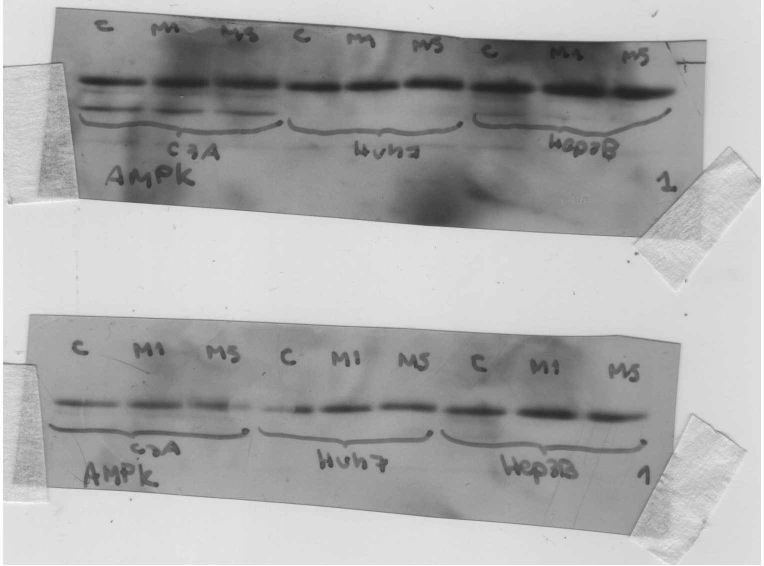

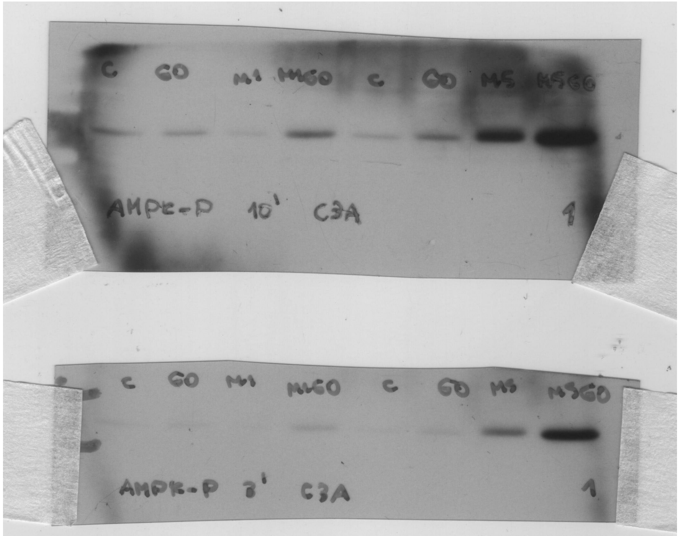

RAW DATA FIG. 5

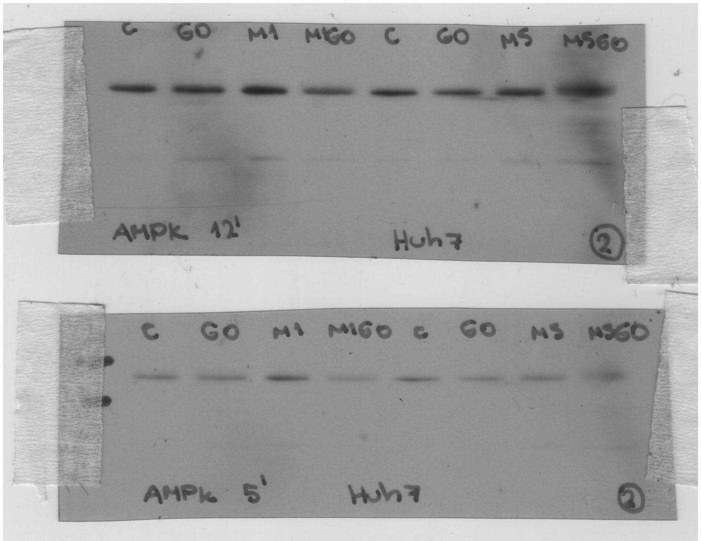

RAW DATA FIG. 5

RAW DATA FIG. 5

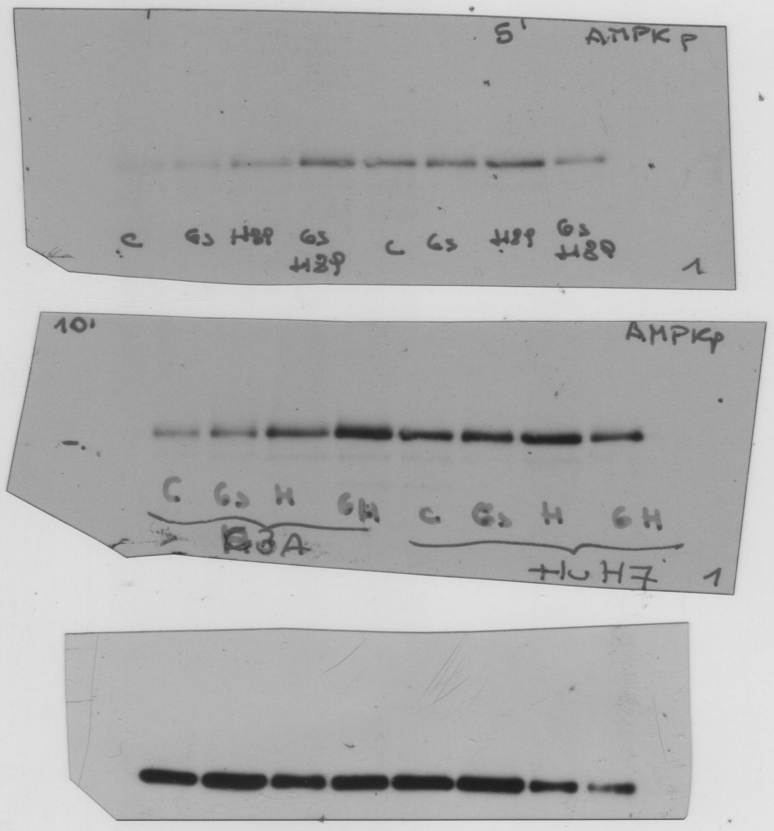

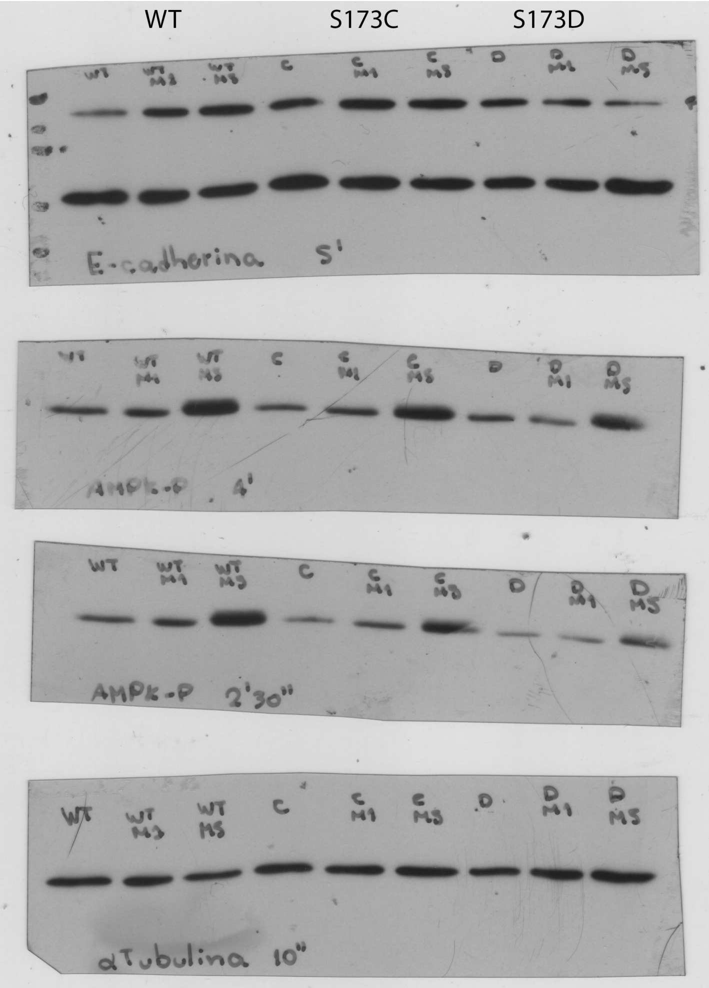

RAW DATA FIG. 6
